# Red-Shifted Epac-Based FRET cAMP Sensors for All-Optical cAMP Control and Multiparameter Imaging

**DOI:** 10.3390/cells15131223

**Published:** 2026-07-06

**Authors:** Tabea Kressmann, Christian Hermann, Aaron Treder, Thomas Gudermann, Ursula Storch, Michael Mederos y Schnitzler

**Affiliations:** 1 Walther Straub Institute of Pharmacology and Toxicology, Ludwig Maximilian University of Munich, 80336 Munich, Germany; 2 Institute of Pharmacy, Clinical Pharmacy, University of Regensburg, 93053 Regensburg, Germany; 3 DZHK (German Centre for Cardiovascular Research), Munich Heart Alliance, 80336 Munich, Germany

**Keywords:** cAMP, dynamic FRET, optogenetic, calcium

## Abstract

**Highlights:**

**What are the main findings?**

- We developed and systematically evaluated four red-shifted Epac-based single-chain FRET biosensors for live-cell cAMP imaging.
- We identified Epac_red4_ as the most sensitive variant with strong responses to cAMP changes.

**What are the implications of the main findings?**

- Epac_red4_ enables multiplex, all-optical signaling studies.
- Epac_red4_ is compatible with blue-light–driven adenylyl cyclase activation and simultaneous readout of cAMP dynamics alongside Ca^2+^ imaging in living cells.

**Abstract:**

Cyclic adenosine monophosphate (cAMP) is a ubiquitous second messenger downstream of G protein-coupled receptors (GPCRs) and a central regulator of cellular signaling. Genetically encoded exchange proteins directly activated by cAMP (Epac)-based Förster resonance energy transfer (FRET) biosensors enable real-time monitoring of cAMP dynamics in living cells, but commonly used cyan/yellow FRET pairs require short-wavelength excitation, limiting compatibility with multiplex imaging and blue-light optogenetic tools such as bacterial photoactivated adenylyl cyclases (bPACs). Here, we engineered and systematically characterized four red-shifted Epac-based single-chain FRET cAMP sensors combining yellow or orange FRET donors with red fluorescent FRET acceptors. Using ratiometric live-cell imaging, we quantified stimulus-evoked FRET responses and identified Epac_red4_ as the best-performing variant, showing an approximately 55% decrease in normalized FRET after forskolin stimulation. Epac_red4_ also reliably detected G_i/o_-mediated decreases in cAMP following μ-opioid receptor activation. Brief 405 nm light pulses induced graded and reversible cAMP elevations using the low dark-activity variant bPAC-F198Y. Furthermore, Epac_red4_ enabled analysis of cAMP recovery kinetics during phosphodiesterase inhibition and multiplex imaging of cAMP and intracellular Ca^2+^ using Fura-2 with minimal spectral and pH-related interference under physiological imaging conditions. Together, Epac_red4_ represents a robust red-shifted cAMP sensor for optogenetic and multiplex signaling studies.

## 1. Introduction

G protein-coupled receptors (GPCRs) constitute the largest family of membrane proteins, transducing a vast array of extracellular signals into specific cellular responses by activating intracellular signal transduction pathways [1,2]. Given their involvement in nearly all physiological and pathophysiological processes and their status as molecular targets for 36% of all approved drugs [3], a high-resolution analysis of GPCR signaling is of utmost importance. A primary effector system regulated by GPCRs is the adenylyl cyclase (AC) family, where intracellular adenosine 3′,5′-cyclic monophosphate (cAMP) levels are tightly regulated by the opposing actions of G_s_ proteins leading to cAMP level increases and G_i/o_ proteins resulting in cAMP level decreases [4]. This regulatory equilibrium is further refined by the enzymatic activity of phosphodiesterases (PDEs), which catalyze the degradation of cAMP into 5′-AMP. By balancing G protein-mediated synthesis with PDE-mediated hydrolysis, the cell achieves precise spatiotemporal control over intracellular cAMP concentrations. This control is fundamentally rooted in the organization of cAMP signaling into highly localized nanodomains, which prevent the uniform diffusion of this second messenger and ensure that specific GPCR inputs trigger distinct cellular responses [5].

To monitor these cAMP fluctuations in living cells, Förster resonance energy transfer (FRET)-based sensors have emerged as a powerful alternative to traditional biochemical or radioactive approaches. These optical sensors typically exploit the conformational changes in the exchange protein directly activated by cAMP (Epac), a family of nucleotide exchange factors discovered in 1998 that revealed a signaling mechanism independent of protein kinase A (PKA) [6,7]. Structurally, Epac-based sensors consist of a regulatory cAMP-binding domain (CNBD) sandwiched between a donor and an acceptor fluorophore. To prevent interference with endogenous signaling and ensure cytosolic diffusion, the membrane-targeting disheveled-EGL-10-pleckstrin (DEP) domain is removed, and the catalytic guanine nucleotide exchange factor (GEF) domain is often inactivated via point mutations.

The evolution of these sensors [8,9,10] has led to highly optimized FRET constructs such as Epac_H187_ [11], which utilizes a monomeric Turquoise2 [12] (mTurquoise2, mTq2) as a FRET donor and the tandem dimer of circularly permuted and monomeric Venus (tdVenus) as a FRET acceptor. Featuring an additional amino acid exchange from glutamine to glutamate at position 270 (Q270E) to increase cAMP affinity, Epac_H187_ currently represents the most responsive blue-shifted FRET-based cAMP sensor and serves as the benchmark for highest sensitivity and dynamic range [10].

Besides FRET-based approaches, several genetically encoded single-fluorescent-protein cAMP sensors have recently expanded the available toolbox for live-cell cAMP imaging. These include yellow/green sensors such as Flamindo2 [13] and G-Flamp1 [14], red-shifted sensors such as Pink Flamindo [15] and R-FlincA [16], and highly sensitive sensors such as cAMPinG1 [17]. In addition, alternative ratiometric cAMP sensors have been developed, including gCarvi [18], which is based on the cAMP-binding domain of the bacterial cAMP receptor protein, and optimized Epac-derived FRET/FLIM sensors such as cAMPFIRE [19]. These sensors differ substantially in their apparent cAMP affinity, dynamic range, spectral properties, and readout principles. Nevertheless, they offer simplified imaging configurations and facilitate multiplex imaging, as cAMP-dependent fluorescence changes can be monitored within a single detection channel. However, unlike FRET-based biosensors, single-fluorophore sensors generally lack an intrinsically ratiometric readout. Consequently, their signals may be more susceptible to variations in sensor expression levels and more strongly affected by photobleaching than those obtained with ratiometric FRET biosensors. In contrast, the intrinsically ratiometric readout of FRET sensors provides an internal normalization that reduces the impact of these experimental factors, thereby facilitating more valid quantitative analysis of intracellular cAMP dynamics [11]. Altogether, single-fluorophore and FRET-based cAMP biosensors represent complementary approaches with distinct advantages depending on the experimental application.

However, the reliance of the established Epac-based FRET cAMP sensor, such as Epac_H187_ [11], on short-wavelength excitation, particularly around 405 nm for mTurquoise2, poses a significant limitation for advanced experimental settings. Shifting the cAMP sensing technology into the red-shifted spectrum is essential for two primary reasons. First, it ensures compatibility with optogenetics, as the activation spectrum of the bacterial photoactivated adenylyl cyclase (bPAC) overlaps significantly with the excitation wavelengths of blue-shifted donors [20]. This spectral interference leads to unintended “leak” activation of bPAC during the FRET readout, compromising the independent control of cAMP levels [20,21]. Second, it enables simultaneous multi-parameter imaging. For complex signaling assays, it is often necessary to monitor cAMP alongside other messengers, such as calcium (Ca^2+^), to understand their intricate cross-talk [22]. For example, the gold-standard ratiometric calcium indicator Fura-2 requires excitation in the UV/violet range (340/380 nm) [23]. Simultaneous monitoring of Ca^2+^ and cAMP using Fura-2 and Cyan/Yellow FRET sensors (e.g., mTurquoise2/Venus) is hampered by dual spectral crosstalk. Not only the UV excitation used for Fura-2 (340/380 nm) partially excites CFP-like donors due to their broad excitation tails, but, even more critically, the broad emission of Fura-2 (~510 nm) significantly overlaps with both the donor (~480 nm) and acceptor (~530 nm) channels, leading to substantial bleed-through. Utilizing a red-shifted cAMP sensor resolves this by shifting the FRET signals into a transparent spectral “window”, enabling crosstalk-free ratiometric imaging of Ca^2+^ and cAMP dynamics within the same single cell.

In this study, we developed and systematically characterized four novel red-shifted Epac-based FRET sensors, Epac_red1_, Epac_red2_, Epac_red3_ and Epac_red4_, that exhibit various combinations of yellow, orange, and red fluorescent proteins. These include a tandem dimer form of LanYFP [24] as well as monomeric variants of Orange2 [25] and Scarlet3 [26]. Our goal was to identify a variant that matches the robustness of the Epac_H187_ benchmark while offering the spectral flexibility required for multi-modal imaging and optogenetic manipulation. We identified Epac_red4_ as a red-shifted lead candidate through pharmacological stimulation and a finely tuned optogenetic approach using a bPAC mutant to demonstrate precise, all-optical control of intracellular cAMP dynamics. Furthermore, we demonstrate that Epac_red4_ is well suited for multiplexed imaging, enabling simultaneous and ratiometric monitoring of intracellular Ca^2+^ and cAMP dynamics.

## 2. Materials and Methods

### 2.1. Chemicals

Poly-L-lysine (Cat. No. P-1524), bovine serum albumin (BSA; Cat. No. A7030), 3-isobutyl-1-methylxanthine (IBMX, Cat. No. I5879) and isoproterenol hydrochloride (Isoprenaline, Cat. No. I6504) were purchased from Sigma-Aldrich (Taufkirchen, Germany). Forskolin (FSK, Cat. No. HY-15371) was obtained from Hölzel Diagnostika Handels GmbH (Köln, Germany). DAMGO (Cat. No. HB2409) was purchased from hellobio (Dunshaughlin, Ireland), and vasoactive intestinal peptide (VIP; Cat. No. 1911) from Bio-Techne GmbH (Wiesbaden-Nordenstadt, Germany). All stock solutions were prepared according to the manufacturers’ instructions, aliquoted, and stored at −20 °C unless stated otherwise. Forskolin was freshly prepared in HEPES-buffered DMEM without phenol red. The end concentration of forskolin is 1 mM for experimental use. For forskolin pre-stimulation with a 1 µM end concentration, a 1 mM stock solution with anhydrous DMSO was prepared, aliquoted, and stored for up to three months. DAMGO was dissolved in double-distilled water to a 200 mM stock solution and used at a final concentration of 200 µM. Isoprenaline was dissolved to a 100 mM stock solution in double-distilled water supplemented with 0.05% sodium metabisulfite (Cat. No. 1.06357, Sigma-Aldrich) to prevent degradation and used at a final concentration of 100 µM. VIP was prepared in double-distilled water to a 10 mM stock solution and used at a final concentration of 10 µM. IBMX was dissolved in anhydrous DMSO to obtain 100 mM and 500 mM stock solutions and used at final concentrations of 100 µM and 500 µM. The final DMSO concentration in all experiments was kept at <2‰. Unless stated otherwise, compounds were applied via bath application at a volume dilution ratio of 1:3, and 10× higher concentrations of maximal effect concentration of receptors, agonists, activators and inhibitors were used to minimize delayed wash-in effects.

### 2.2. Cell Lines and Culture Conditions

Human embryonic kidney (HEK293T) cells (Leibniz-Institute DSMZ, Braunschweig, Germany; ACC 635) were employed for all experiments. HEK293T cells were maintained in Earl’s Minimal Essential Medium (MEM; Sigma-Aldrich, Taufkirchen, Germany) supplemented with 10% (*v*/*v*) fetal calf serum (FCS; Gibco, Life Technologies, Carlsbad, CA, USA), 100 U/mL penicillin, and 100 μg/mL streptomycin. HEK29T cells were maintained at 37 °C in a humidified atmosphere containing 5% CO_2_.

### 2.3. FRET-Based cAMP Sensor Architecture

The cytosolic cAMP FRET sensor mTurquoise2-Epac^Q270E^-cp173mVenus-cp173mVenus [11] served as the parental construct. To maintain consistency with existing literature while defining a clear nomenclature for this study, this sensor—frequently cited as H187—is referred to as Epac_H187_ throughout this work. The Epac_H187_ backbone features a deleted membrane-targeting DEP domain (ΔDEP) and is catalytically inactive due to two point mutations (T781A and F782A) [27] within the guanine nucleotide exchange factor (GEF) domain, preventing unintended Ras-related protein (Rap) activation. Additionally, a high-affinity amino acid substitution (Q270E) in the cyclic nucleotide-binding domain (CNBD) was utilized to enhance sensitivity toward cAMP. Note that amino acid numbering for all Epac mutations follows the nomenclature of the original Epac-based sensor characterization [11], independent of the N-terminal fluorophore tags.

### 2.4. Design and Generation of Red-Shifted Sensor Variants

Four novel red-shifted sensor variants, designated Epac_red1_ through Epac_red4_, were developed through systematic fluorophore replacement. Primers for site-directed mutagenesis and Gibson assembly were designed using NEBaseChanger and NEBuilder Assembly Tool (New England Biolabs, Ipswich, MA, USA), respectively, and synthesized by Sigma-Aldrich (desalted, 100 μM). Detailed primer sequences and corresponding annealing temperatures are summarized in Table 1. The first red-shifted variant, Epac_red1_ (tdV-Epac-cpmCh2), was generated by replacing the N-terminal mTurquoise2 with circularly permuted, monomeric Cherry2 (cpmCherry2; #52100, Addgene, Watertown, MA, USA) via PCR-based deletion and subsequent ligation, yielding the configuration cp173mVenus-cp173mVenus-Epac^Q270E^-cpmCherry2. To create the Epac_red2_ (mOrange2-Epac-cpmCh2) variant, both cp173mVenus units were excised and replaced by incorporating monomeric Orange2 (mOrange2; Addgene #54568) at the N-terminus. For the Epac_red3_ (tdOrange2-Epac-tdSc3) construct, tandem dimer Scarlet3 (tdScarlet3) was fused to the C-terminus. The mScarlet3 sequence (Addgene #189767) was modified by removing the LifeAct tag and introducing N-terminal NheI and C-terminal EcoRI restriction sites. Following PCR amplification and ligation of two mScarlet3 units, the resulting tdScarlet3 was inserted into the backbone. The N-terminal tandem dimer Orange2 (tdOrange2) was generated by codon-optimizing mOrange2, adding a flexible linker, and fusing it to a second mOrange2 unit before insertion into the EcoRV-digested backbone. Finally, the Epac_red4_ (tdLanYFP-Epac-tdSc3) sensor was produced using tandem dimer LanYFP (tdLanYFP) synthesized by GenScript (Piscataway, NJ, USA). The fragment was cloned into the pcDNA3.1(+) vector and subsequently fused to the N-terminus of the Epac backbone via EcoRV digestion.

### 2.5. Co-Expression Strategies and Optimization

To enable optogenetic control, the red-shifted sensors were co-expressed with the bacterial photoactivated adenylyl cyclase (bPAC). Initially, an internal ribosome entry site (IRES) sequence was utilized downstream of the sensor coding sequence. For the lead candidate Epac_red4_ (tdLanYFP-Epac-tdSc3), bPAC was further optimized by introducing the F198Y point mutation. To ensure precise 1:1 stoichiometry and robust expression levels, the IRES sequence was ultimately replaced with a P2A self-cleaving peptide [28] via PCR-based cloning.

### 2.6. Imaging Preparation and Transfection

For FRET experiments, cells were seeded onto glass-bottom dishes (FluoroDish, 35 mm diameter, 23 mm glass bottom; WPI, Friedberg, Germany). Prior to seeding, dishes were coated at room temperature with 0.5 mL poly-L-lysine (0.1 mg/mL; Sigma-Aldrich) for 30 min. After removal of the coating solution, dishes were washed once with 2 mL sterile DPBS (Sigma-Aldrich), and cells were seeded in their respective maintenance media. HEK293T cells were transfected with 0.5 μg of the indicated Epac sensor construct, with or without IRES- or -P2A-mediated co-expression of bPAC, using the non-lipid-based reagent GeneJuice^®^ (Merck Millipore, Schwalbach, Germany) according to the manufacturer’s instructions. Where indicated, 1 μg of the human μ-opioid receptor (µOR, pcDNA 3.1+, cDNA Resource Center, #OPRM100000), the human adrenergic β_2_ receptor (β_2_R, pcDNA 3.1+, cDNA Resource Center, #AR0B200000), or the vasoactive intestinal peptide receptor 1 (VPAC1R, cDNA Resource Center, #VIPR100000) was co-transfected. All measurements were performed approximately 48 h post-transfection.

### 2.7. Live-Cell FRET Imaging

Dynamic changes in intracellular cAMP concentrations were quantified in single living cells using the developed red-shifted Epac-based sensors. Imaging was performed at room temperature (22 ± 2 °C) using a laser-coupled, camera-based microscopy system (iMIC2.0, TILL Photonics/FEI, Gräfelfing, Germany) equipped with a Sole-6 Laser Line Combiner (Omicron Laserage Laserprodukte GmbH, Rodgau-Dudenhofen, Germany). This system featured five integrated laser lines: two diode lasers (405 nm/120 mW and 488 nm/100 mW) and three Diode-Pumped Solid-State (DPSS) lasers (515 nm/100 mW, 561 nm/100 mW, and 594 nm/100 mW). The laser lines were coupled into the iMIC2 microscope without the use of an automated fiber switcher to ensure maximum power stability. To virtually eliminate thermal noise and ensure a stable baseline independent of ambient temperature fluctuations, the internal multi-stage Peltier cooling of the back-illuminated EMCCD camera (iXon3 897, model DU-897U-CS0; Andor Technology, Belfast, UK; 512 × 512-pixel sensor with a pixel size of 16 μm) was supplemented with an external water-circulating heat exchanger (LAUDA, Lauda-Königshofen, Germany). The cooling water was maintained at a constant temperature of +14.0 ± 0.1 °C, enabling the EMCCD chip to operate at a highly stable temperature of −95 °C To prevent moisture condensation and ensure stable optical performance at these cryogenic temperatures, the camera port was specifically modified with a custom-built, perforated dehumidification chamber containing a mixture of molecular sieve (5 Å, Carl Roth, Karlsruhe, Germany) and indicator silica gel (red/yellow, grain size 1–3 mm, Carl Roth). Additionally, the interior of the microscope body was dehumidified using permeable pouches filled with the same compounds as those used in the chamber, strategically placed to ensure an unobstructed optical path and unimpeded mechanical movement.

### 2.8. Image Acquisition, Region of Interest (ROI) Analysis and FRET Quantification

Images were acquired using Live Acquisition software (version 2.5.0.15, TILL Photonics/FEI) at a frequency of 1 Hz with an exposure time of 10 ms per channel. To accommodate filter changes, appropriate switching times were included between channel acquisitions. Camera settings consisted of an EM gain of 20 and 2 × 2-pixel binning to enhance the signal-to-noise ratio. Cells were imaged using a 40× oil-immersion objective (UApo N340, N.A. 1.35, W.D. 0.10 mm; Olympus, Tokyo, Japan). To minimize photobleaching prior to measurements, cell identification and morphology-based selection were performed using low-intensity 594 nm excitation (10%). Fluorophores were excited sequentially using specific laser lines with intensities adjusted for each sensor: mTurquoise2 was excited at 405 nm (100% intensity), while tdVenus, mOrange2, tdOrange2, and tdLanYFP were excited at 515 nm (10–20% intensity). For the red-shifted acceptors, cpmCherry2 was excited at 594 nm (10%), whereas tdScarlet3 was excited at either 561 nm (10%) when paired with tdLanYFP or at 594 nm (60%) when paired with tdOrange2. Emission signals were separated using a 585 nm dichroic beamsplitter.

For data quantification, a single region of interest (ROI) was manually defined for each fusiform cell. These ROIs were strictly confined to the cytoplasmic compartment, carefully excluding the nucleus to ensure a purely cytosolic FRET readout. Depending on individual cell morphology, ROI dimensions typically ranged from 8 to 20 binned pixels (using the 40× objective). Only cells displaying fluorescence intensities 3- to 10-fold above the background (determined in a cell-free area of the imaging field) were included in the analysis.

To account for non-specific signals, mean background intensities measured in the donor (F_Donor_), acceptor (F_Acceptor_), and FRET (F_FRET_) channels were subtracted from the corresponding raw fluorescence intensities. To correct for optical cross-talk, including spectral bleed-through and cross-excitation, normalized FRET (N_FRET_) was calculated using a custom-written script in R (version 4.2.3) based on the method described by Xia and Liu (2001) [29]. Bleed-through coefficients (a and b) were determined using HEK293T cells expressing either the donor or acceptor alone. N_FRET_ was calculated by subtracting donor and acceptor contributions from the FRET channel and normalizing to the geometric mean of donor and acceptor fluorescence intensities.

$$N_{FRET}=\frac{F_{FRET}-aF_{Donor}-bF_{Acceptor}}{\sqrt{F_{Donor}\times F_{Acceptor}}}$$

Subsequently, N_FRET_ signals were normalized to baseline by dividing each value by the geometric mean of N_FRET_ (N_FRETgeom_) values recorded between 10 s and 50 s.

$${N_{FRET}}_{geom}=\sqrt[{n}]{\prod_{i=10\;s}^{50\;s}N_{FRET}(t_{i})}$$

$$N_{FRET}^{norm}\left(t\right)=\frac{N_{FRET}(t)}{{N_{FRET}}_{geom}}$$

Epac_red_ sensor signals can also be expressed as the simple ratio F_FRET_/F_Donor_; however, this representation is used exclusively for real-time visualization in the LA software, (version 2.5.0.15) enabling qualitative monitoring and comparison of responses during acquisition. All quantitative analyses in this study are based on N_FRET_ values corrected according to Xia and Liu (2001) [29]. A decrease in normalized N_FRET_ corresponds to an increase in intracellular cAMP levels, reflecting the conformational change in the Epac sensor upon ligand binding. Further data processing, including baseline normalization and statistical analysis, was performed using OriginPro 2026 (OriginLab, Northampton, MA, USA).

### 2.9. Simultaneous Dual-Parameter Imaging of cAMP and Ca^2+^

To investigate the spatio-temporal relationship between cAMP and Ca^2+^ signaling, simultaneous imaging experiments were performed. Intracellular Ca^2+^ levels were monitored using the ratiometric indicator Fura-2. Cells were loaded with 5 µM Fura-2-acetoxymethyl ester (Fura-2 AM, Invitrogen/Thermo Fisher Scientific, Waltham, MA, USA) in HEPES-buffered Hanks’ Balanced Salt Solution (HBSS) containing 0.1% BSA and 0.04% Pluronic^®^ F-127 for 30 min at 37 °C in a humidified atmosphere containing 5% CO_2_, followed by three wash steps with 2 mL HBSS each to allow for complete de-esterification of the dye.

Dual-parameter imaging was achieved by synchronizing the laser-based iMIC2.0 FRET setup with a Polychrome V illumination system (TILL Photonics/FEI, Munich, Germany) equipped with a 150 W xenon arc lamp. Cells were imaged using a 20× UV-transmissive oil-immersion objective (UPlanSApo 20× Oil, N.A. 0.85, W.D. 0.20 mm; Olympus, Tokyo, Japan; system FN 26.5) mounted on the iMIC system, which enabled both Fura-2 and FRET measurements. Fura-2 fluorescence was excited alternately at 340 nm and 380 nm, and emission was collected at 510 nm. To prevent spectral overlap between the Fura-2 emission and the FRET donor signal, a specialized multiband dichroic beamsplitter and high-performance emission bandpass filters (510/20 nm for Fura-2 and 470/24 nm for mTurquoise2) were utilized.

Acquisition was strictly interleaved, ensuring that the UV excitation for Fura-2 (340/380 nm) and laser excitation for FRET were performed in rapid succession to avoid simultaneous fluorescence emission and minimize crosstalk. To further evaluate potential spectral crosstalk, the excitation spectra of the employed fluorophores were considered during experimental design. While tdLanYFP exhibits an excitation maximum in the green spectral range at 513 nm, excitation at 340 nm was not detectable under our imaging conditions, and excitation at 380 nm was negligible. Conversely, Fura-2 fluorescence was exclusively recorded during UV excitation periods and detected through dedicated emission filters centered at 510 nm. Together with the sequential acquisition protocol, this ensured effective optical and temporal separation of the cAMP and Ca^2+^ signals.

Crucially, identical ROIs were used for FRET and Fura-2 analysis to ensure precise spatial co-registration of both second messengers. A single cytoplasmic ROI per cell was selected, excluding the nucleus, and typically comprised 5–12 binned pixels depending on cell morphology (using the 20× objective). Acquisition was interleaved with the FRET protocol, ensuring near-simultaneous recording of both second messengers. According to the Grynkiewicz method (1985) [23], intracellular Ca^2+^ concentrations were expressed as the ratio of fluorescence intensities (R = F_340_/F_380_). Background subtraction was performed for both wavelengths (340 nm and 380 nm) prior to ratio calculation. This combined optical and temporal separation ensured a crosstalk-free, near-simultaneous recording of both second messengers under stable physiological conditions. Measurements were performed in HEPES-buffered saline (HBS) solution containing 140 mM NaCl, 5.4 mM KCl, 1 mM MgCl_2_, 2 mM CaCl_2_, 10 mM glucose, and 10 mM HEPES (pH 7.4 adjusted with NaOH; osmolarity 295–302 mOsm kg^−1^).

### 2.10. Quantification and Statistical Analysis

Statistical analyses were performed in Origin 2026 (OriginLab Corporation, Northampton, MA, USA) using an unpaired Mann–Whitney U test, Kruskal–Wallis test, paired Wilcoxon matched-pairs signed-rank test followed by post hoc Dunn’s test, or Friedman test followed by post hoc Dunn’s test where appropriate. A *p* value < 0.05 was considered statistically significant. Significance levels were defined as * *p* < 0.05, ** *p* < 0.01, and *** *p* < 0.001. Box plots represent the median and interquartile range. The specific statistical tests used are indicated in the respective figure legends.

## 3. Results

### 3.1. Structural Design and Spectral Characterization of Red-Shifted Epac-Based FRET Sensors

To expand the toolkit for multicolor imaging, we developed four red-shifted cAMP FRET sensors (summarized in Table 2) by fusing various donor and acceptor fluorophores to the N- and C-termini of the Epac sensory domain. As a reference, we utilized the established blue-shifted sensor Epac_H187_, which features an N-terminal monomeric Turquoise2 (mTq2) as the FRET donor and a C-terminal tandem dimer of circularly permuted monomeric Venus (tdV) as the FRET acceptor [11]. The red-shifted variants were designed with increasing theoretical FRET efficiencies. In the first two constructs, Epac_red1_ and Epac_red2_, circularly permuted and monomeric Cherry2 (cpmCherry2) was fused to the C-terminus to serve as the FRET acceptor, and either tandem dimer Venus or monomeric Orange2 (mOr2) was fused to the N-terminus to serve as the FRET donor. For the third variant, Epac_red3_, we employed tandem dimer Orange2 (tdOr2) as the FRET donor at the N-terminus and tandem dimer Scarlet3 (tdSc3) as the FRET acceptor at the C-terminus. Excitation for the three sensors, Epac_red1_, Epac_red2_ and Epac_red3_, was performed by applying Laser light of the wavelengths 515 nm for donor excitation and 594 nm for acceptor excitation. Finally, we generated Epac_red4_, incorporating tandem dimer LanYFP (tdLanYFP) as an N-terminal FRET donor and tandem dimer Scarlet3 as a C-terminal FRET acceptor, which were excited using light of the wavelengths 515 nm and 561 nm, respectively. Notably, the sensors utilizing tandem dimer Scarlet3 as the C-terminal FRET acceptor (Epac_red3_ and Epac_red4_) exhibited the highest calculated FRET efficiencies (R_0_ × QY_A_) of 47 and 50, respectively (calculated using https://www.fpbase.org/fret/ (accessed on 15 January 2026)).

### 3.2. Functional Validation of Red-Shifted cAMP Sensors

**Figure 1:**
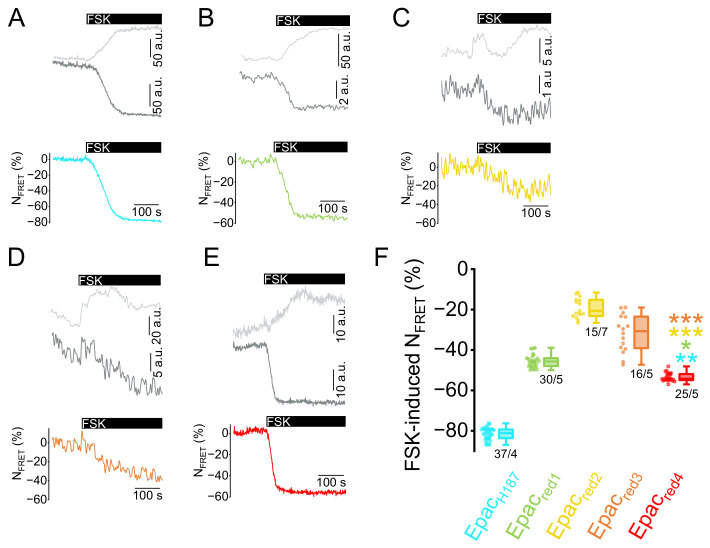
Functional validation and dynamic range of red-shifted cAMP FRET sensors. (**A**–**E**) Representative single-cell recordings of HEK293T cells expressing the established blue-shifted FRET-based cAMP sensor Epac_H187_ (**A**) or the red-shifted variants Epac_red1_ (**B**), Epac_red2_ (**C**), Epac_red3_ (**D**), and Epac_red4_ (**E**). Upper panels: Cross-talk-corrected fluorescence intensity traces of the respective donor and acceptor fluorophores. Lower panels: Calculated normalized FRET (N_FRET_) traces. Gray bars indicate pharmacological stimulation with the adenylyl cyclase activator forskolin (FSK, 1 mM). (**F**) Summary of FSK-induced maximal N_FRET_ signals. Data are presented as box-and-whisker plots (median, interquartile range, and min/max whiskers) with individual data points (color-coded dots) plotted to the left of each box. The numbers below each box indicate the total number of analyzed cells and the number of independent transfections. Color-coded asterisks indicate significance levels (* *p* < 0.05, ** *p* < 0.01, *** *p* < 0.001, Mann–Whitney U-test) for the comparison of Epac_red4_ against the other cAMP sensors.

To evaluate the functionality and dynamic range of the newly developed red-shifted cAMP sensors, we performed live-cell dynamic FRET imaging in HEK293T cells overexpressing the cAMP FRET sensors. Intracellular cAMP levels were maximally elevated by pharmacological stimulation with the adenylyl cyclase activator forskolin (FSK, 1 mM). Representative single-cell recordings for each sensor are shown in Figure 1A–E (upper panels) as cross-talk-corrected, time-resolved fluorescence traces. Forskolin stimulation induced reciprocal changes in the individual fluorescence channels, characterized by an increase in donor fluorescence and a concomitant decrease in acceptor fluorescence. These opposing responses resulted in a robust decrease in the calculated FRET signal. FRET changes were quantified as normalized FRET (N_FRET_) to account for spectral bleed-through and variations in fluorophore expression levels. All cAMP sensors were functional, showing FRET signal decreases induced by the application of forskolin (Figure 1A–E, lower panels). Notably, Epac_red4_ provided a slightly higher signal-to-noise ratio and more consistent kinetics than Epac_red1_, as seen in the individual cell N_FRET_ traces (Figure 1B,E, lower panels). However, the summary of the forskolin-induced FRET signal changes revealed pronounced differences in the response of the various cAMP sensors to this pharmacological intervention (Figure 1F). The well-established blue-shifted sensor Epac_H187_ (mTq2-Epac-tdV, ref. [11]) served as a benchmark, exhibiting the most robust response with a mean decrease in N_FRET_ of approximately 80%, which confirms its high dynamic range and reliability.

Among the red-shifted candidates, Epac_red4_ (tdLanYFP-Epac-tdSc3) and Epac_red1_ (tdV-Epac-cpmCh2) emerged as the most effective variants under these pharmacological conditions, with Epac_red4_ showing a significantly larger N_FRET_ decrease (approximately 55%) than Epac_red1_ (approximately 45%) and all other red sensors (Figure 1F, color-coded asterisks). While this demonstrates superior dynamic range within the red-shifted group, the response of Epac_red4_ was significantly lower compared to the maximal ΔN_FRET_ amplitude of the blue-shifted benchmark Epac_H187_ (Figure 1F, cyan asterisks).

To characterize the spectral properties of the fluorescent proteins incorporated into the Epac-based cAMP FRET sensors, we performed live-cell fluorescence spectroscopy in transiently transfected HEK293T cells (see Supplementary Methods). Excitation and emission spectra were recorded as background-corrected difference spectra by subtracting the autofluorescence signals of untransfected cells (Supplementary Figure S1). Quantitative analysis demonstrated that the experimentally determined excitation and emission maxima were in good overall agreement with published reference values obtained from FPbase (https://www.fpbase.org/ (accessed on 1 February 2026); Supplementary Table S2). For most fluorophores, deviations between the measured and reference peak wavelengths were ≤2 nm, confirming that the fluorescent proteins retained their expected spectral properties under live-cell conditions. Larger deviations were observed for tdVenus emission and cpmCherry2 excitation. As all measurements were performed in living HEK293T cells expressing intact fusion constructs rather than purified proteins, these differences most likely reflect the influence of the intracellular environment, such as the cytoplasmatic pH. Detailed spectral acquisition settings are summarized in Supplementary Table S1.

Because the quantitative analyses of the Epac sensors throughout this study are based on normalized FRET (N_FRET_), which corrects for spectral bleed-through and cross-excitation, we additionally analyzed the uncorrected (“raw”) FRET ratios (acceptor emission intensity divided by donor emission intensity) derived directly from the background-subtracted emission spectra (Supplementary Figure S2). Although these raw spectral ratios differ numerically from N_FRET_ values because no correction of bleed-through and cross-excitation was applied, they reflect the same ranking of sensor performance observed in the live-cell N_FRET_ measurements (see Figure 1F). In particular, Epac_red4_ exhibited the largest spectral FRET change following stimulation with forskolin and IBMX, independently confirming its superior dynamic range and validating the conclusions drawn from the N_FRET_ analysis.

### 3.3. Optogenetic Validation and Selection of the Lead Red-Shifted cAMP FRET Sensor

**Figure 2:**
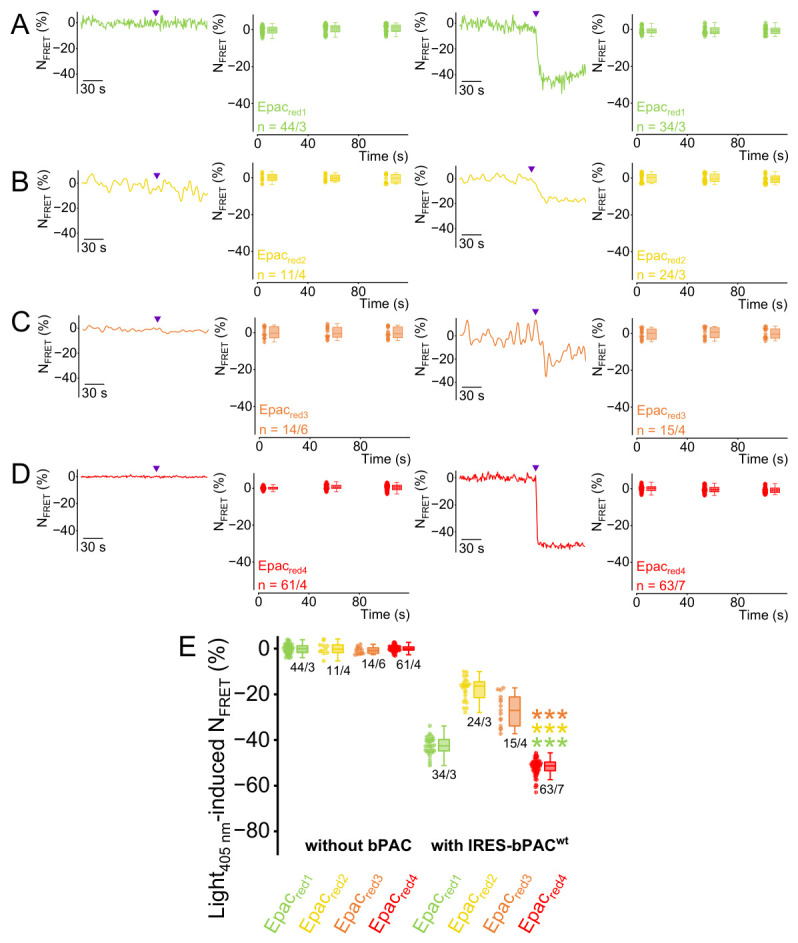
Optogenetic validation of the red-shifted cAMP FRET sensors. (**A**–**D**) Representative N_FRET_ traces of HEK293T cells expressing the red-shifted sensor variants Epac_red1_ (**A**), Epac_red2_ (**B**), Epac_red3_ (**C**), or Epac_red4_ (**D**). (**A**–**D**) First and third panels show N_FRET_ time courses in the absence (first) or presence (third) of co-expressed bacterial photoactivated adenylyl cyclase (bPAC). The second and fourth panels show analysis of N_FRET_ values at three different time points prior to application of a single 405 nm laser light pulse (100 ms). bPAC that was co-expressed using a bicistronic pIRES2 expression vector. Purple arrows indicate a single 405 nm laser light pulse (100 ms) to trigger cAMP production by stimulation of bPAC. (**E**) Summary of the N_FRET_ changes induced by light stimulation. Data are shown as box-and-whisker plots (median, interquartile range, and min/max whiskers) with individual data points plotted to the left of each box. Responses are shown for sensors expressing the red-shifted Epac sensors alone (without bPAC) or co-expressing bPAC (with IRES-bPAC^wt^). The numbers below each box represent the total number of analyzed cells and the number of independent transfections. Color-coded asterisks indicate significance levels (*** *p* < 0.001, Mann–Whitney U-test) for the comparison of Epac_red4_ against the other red-shifted variants.

To complement the pharmacological intervention, we employed an optogenetic approach to evaluate the FRET sensor performance under rapid, light-triggered cAMP dynamics. For this, we utilized the bacterial photoactivated adenylyl cyclase (bPAC), which was co-expressed with the red-shifted cAMP FRET sensors via a bicistronic internal ribosome entry site (IRES)-based plasmid to ensure constant stoichiometric expression. This setup enabled precise, transient elevation of intracellular cAMP levels via a 405 nm laser pulse that was applied for 100 ms to stimulate cAMP production via bPAC. Notably, the blue-shifted reference cAMP FRET sensor Epac_H187_ was excluded from this series, as its excitation spectrum significantly overlaps with the activation range of bPAC, a spectral interference that would compromise the independence of sensor readout and optogenetic control. As shown in Figure 2, laser pulses with 405 nm did not induce non-specific FRET signal changes in control cells lacking bPAC, confirming that the cAMP FRET sensors are photostable and unresponsive to the light trigger itself (Figure 2A–D, first and second panels). However, upon co-expression with bPAC, the same laser pulses with 405 nm triggered a rapid and robust decrease in N_FRET_ across all variants of red-light-shifted cAMP FRET sensors (Figure 2A–D, third panels). The basal N_FRET_ values remained remarkably stable even when bPAC was co-expressed via the IRES-based expression vector system, in which the downstream bPAC cistron is expected to be expressed at substantially lower levels than the upstream Epac sensor (Figure 2A–D, second and fourth panels).

The statistical analysis of these responses (Figure 2E) confirms that performance varied significantly among the red-light-shifted cAMP FRET constructs, mirroring the trends observed upon pharmacological stimulation with forskolin. Epac_red4_ (tdLanYFP-Epac-tdScarlet3) exhibited the most pronounced and consistent decrease in N_FRET_, significantly outperforming the other variants in both signal amplitude and signal-to-noise ratio (*p* < 0.001). Due to its superior dynamic range in both pharmacological and optogenetic assays, Epac_red4_ was selected as the primary red-shifted sensor for all subsequent applications.

### 3.4. bPAC^F198Y^ Is Best Suitable to Stimulate Graded Light-Induced cAMP Increases

To refine our optogenetic toolkit, we directly compared the performance of wild-type bPAC (bPAC^wt^) and the F198Y mutant (bPAC^F198Y^) [30]. Both variants were now linked to the Epac_red4_ sensor via a P2A peptide to ensure equimolar expression levels. While the P2A-based constructs generally exhibited robust expression [28], we observed critical differences in their dark activity, which is the enzyme’s constitutive, light-independent activity.

**Figure 3:**
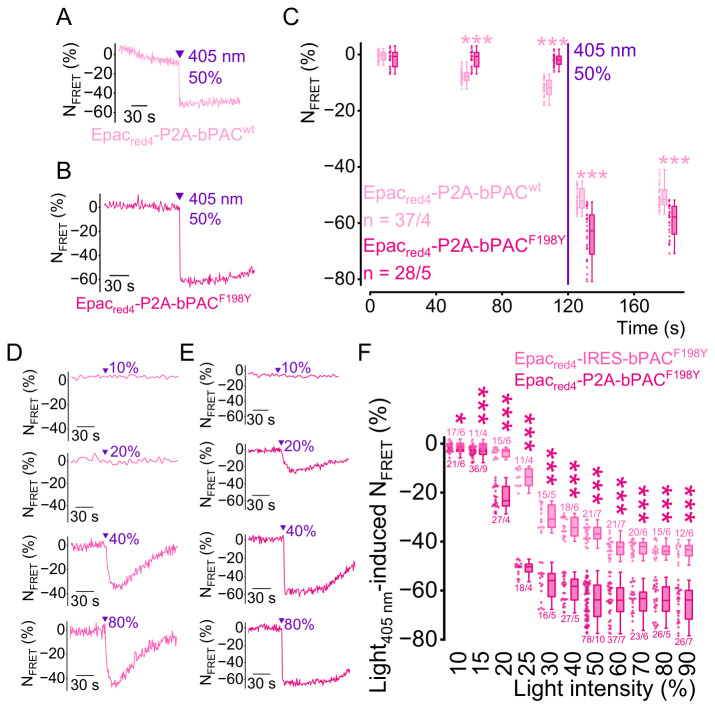
bPAC^F198Y^ enables precise, graded control of cAMP levels with minimal dark activity. (**A**,**B**) Representative N_FRET_ traces of HEK293T cells expressing Epac_red4_ together with bPAC^wt^ (**A**) or bPAC^F198Y^ (**B**). Stoichiometric expression was enabled via a P2A peptide. Purple arrowheads indicate a 405 nm laser light pulse (100 ms, 50% intensity). The continuous baseline decline in (**A**) indicates high dark activity of bPAC^wt^. (**C**) Statistical comparison of N_FRET_ levels at different time points before and after light stimulation in the presence of bPAC^wt^ or bPAC^F198Y^. Significant differences between bPAC^wt^ and bPAC^F198Y^ at each time point were calculated using Mann–Whitney U-test (pink asterisks; *** *p* < 0.001). (**D**,**E**) Representative N_FRET_ traces showing the graded response of Epac_red4_ to 405 nm light pulses with increasing light intensities from 10% to 80% using cDNA expression vectors that contain either an IRES (**D**) or P2A sequence (**E**) for co-expression of Epac_red4_ and bPAC^F198Y^. (**F**) Summary of the maximal N_FRET_ signals induced by application of increasing light intensities (10–90%). Data are presented as box-and-whisker plots (median, interquartile range, and min/max whiskers) with individual data points plotted to the left of each box. The numbers below each box represent the total number of analyzed cells and number of independent transfections. Asterisks (* *p* < 0.05, *** *p* < 0.001, Mann–Whitney U-test) indicate significant differences between the N_FRET_ signals at the indicated light intensities obtained by using the P2A (magenta) or the IRES (green) construct.

Cells expressing bPAC^wt^ frequently exhibited a gradual, light-independent decline in N_FRET_ prior to photostimulation, whereas cells expressing bPAC^F198Y^ maintained a stable baseline. This behavior is consistent with the reduced dark activity previously reported for the F198Y mutant (Figure 3A). Notably, a comparable baseline decline was not observed when bPAC^wt^ was co-expressed using the IRES-based vector system (Figure 2D,E). This difference is likely explained by the distinct expression strategies: whereas P2A-mediated co-expression is expected to result in near-equimolar expression of Epac_red4_ and bPAC, IRES-dependent expression typically results in substantially lower expression of the downstream cistron. Consequently, the dark activity of bPAC^wt^ became more apparent under the P2A-based expression conditions.

In contrast, the bPAC^F198Y^ variant exhibited markedly reduced constitutive activity, maintaining a stable baseline until triggered by a laser pulse (Figure 3B). This distinction is further highlighted by the temporal analysis at specific intervals (e.g., after 60 and 110 s) (Figure 3C), where the bPAC^wt^ variant leads to a significant pre-pulse decline in N_FRET_ compared to the stable baseline of the F198Y variant. Upon stimulation with a 405 nm light pulse at 120 s, both variants showed a rapid increase in intracellular cAMP levels, reflected by a sharp decline in the N_FRET_ signal (Figure 3A,B). Notably, this light-induced cAMP elevation was significantly more pronounced in cells expressing the bPAC^F198Y^ mutant at both 130 s and 180 s compared to bPAC^wt^ (Figure 3C), highlighting the mutant’s superior dynamic range and light-sensitivity.

We further investigated whether bPAC^F198Y^ activity could be modulated in a graded manner by varying the trigger light intensity. Using the Epac_red4_ sensor as a readout, individual cells were stimulated with a 100 ms pulse of 405 nm laser light at intensities ranging from 10% to 90% (Figure 3D,E). The summary of the light-induced FRET signal decreases suggests that the cAMP production is finely tunable, applying 405 nm with increasing light intensities (Figure 3F). At low laser intensities (10–20%), only a slight N_FRET_ decrease was observed, whereas a half-maximal response was achieved at approximately 25% intensity. The FRET signal reached a plateau starting at 50% intensity, indicating a saturation point beyond which further increases in laser power yielded no additional cAMP production. Notably, the comparison between cDNA constructs using IRES or P2A as coupling strategies (Figure 3F) revealed that the P2A-linked bPAC^F198Y^ (magenta) provides a significantly higher dynamic range and more consistent dose–response curves compared to the IRES-coupled version (green). Consequently, the Epac_red4_-P2A-bPAC^F198Y^ construct represents the most effective tool for precise optogenetic cAMP manipulation, offering minimal background activity and superior sensitivity.

### 3.5. Reversibility and Robustness of bPAC^F198Y^-Mediated cAMP Signaling

**Figure 4:**
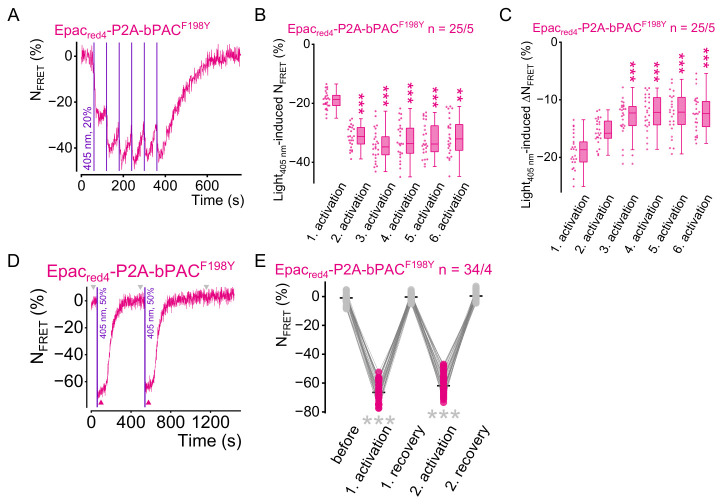
Temporal resolution, reproducibility, and reversibility of bPAC^F198Y^-mediated cAMP signaling. (**A**) Representative N_FRET_ trace of a HEK293T cell expressing the Epac_red4_-P2A-bPAC^F198Y^ construct. The cell was subjected to six consecutive 405 nm laser light pulses (100 ms duration, 20% intensity) at 60 s intervals (indicated by purple vertical lines). (**B**,**C**) Summary of the N_FRET_ responses obtained by the repetitive stimulation protocol shown in (**A**). (**B**) Maximal N_FRET_ decreases achieved after each individual light pulse. (**C**) Individual ΔN_FRET_ decreases induced after each individual light pulse. Asterisks indicate significant differences compared to the first pulse (1. activation; *** *p* < 0.001, Mann–Whitney U-test). (**D**) Representative N_FRET_ trace showing the response to two successive saturating 405 nm laser pulses (100 ms, 50% intensity), demonstrating full reversibility and return to baseline over an extended recovery period. (**E**) Summary of the N_FRET_ signals before, during and after the repetitive saturating stimuli. Individual data points (gray dots and pink dots) represent N_FRET_ signals of individual cells, with gray lines connecting the measurements of each cell across the different states (baseline, activation, recovery). Asterisks indicate significant N_FRET_ changes compared to the respective preceding baseline (** *p* < 0.01, *** *p* < 0.001, Mann–Whitney U-test). The numbers indicate the total number of measured cells and the number of independent transfections.

To assess the temporal resolution and reversibility of the selected Epac_red4_-P2A-bPAC^F198Y^ construct, we performed repetitive optogenetic stimulation assays. Cells were subjected to a series of six consecutive brief 405 nm laser pulses (100 ms duration, every 60 s) at a low, non-saturating intensity of 20% (Figure 4A). Each individual light pulse triggered a rapid decrease in N_FRET_, followed by partial recovery within the one-minute inter-pulse interval. Upon cessation of the stimulation protocol, the N_FRET_ signal returned completely to the baseline. Within 60 s after light stimulation, the FRET signal rapidly decreased and only partially recovered (Figure 4A). Notably, the initial light stimulus induced a significantly smaller maximal N_FRET_ decrease compared with the subsequent repetitive light pulses (Figure 4B). From the second pulse onward, however, the magnitude of the N_FRET_ responses remained remarkably stable, indicating steady-state conditions (Figure 4B). Consistently, the individual ΔN_FRET_ values evoked by repeated light stimulation also remained stable from the third activation onward (Figure 4C). This suggests a priming effect or a rapid reach of a steady state between cAMP production and degradation after the initial stimulus. To challenge the system under maximal load, we applied two successive saturating pulses with a 50% light intensity and observed an extended recovery period (Figure 4D). Under these conditions, the N_FRET_ signal decreased by approximately 65%, reaching a distinct plateau before returning to the baseline within roughly 400 s (Figure 4D). Statistical analysis of these repetitive saturating stimuli (Figure 4E) confirms that both the magnitude of the cAMP response and the kinetics of its recovery are highly reproducible (*p* < 0.001). These data underscore that the Epac_red4_-P2A-bPAC^F198Y^ system enables not only the titratable control of intracellular cAMP levels but also allows for long-term, repetitive measurements of cAMP dynamics with high temporal precision and full reversibility.

### 3.6. Monitoring G_s_- and G_i/o_-Mediated cAMP Signaling Using the Red-Shifted Epac_red4_ Sensor

**Figure 5:**
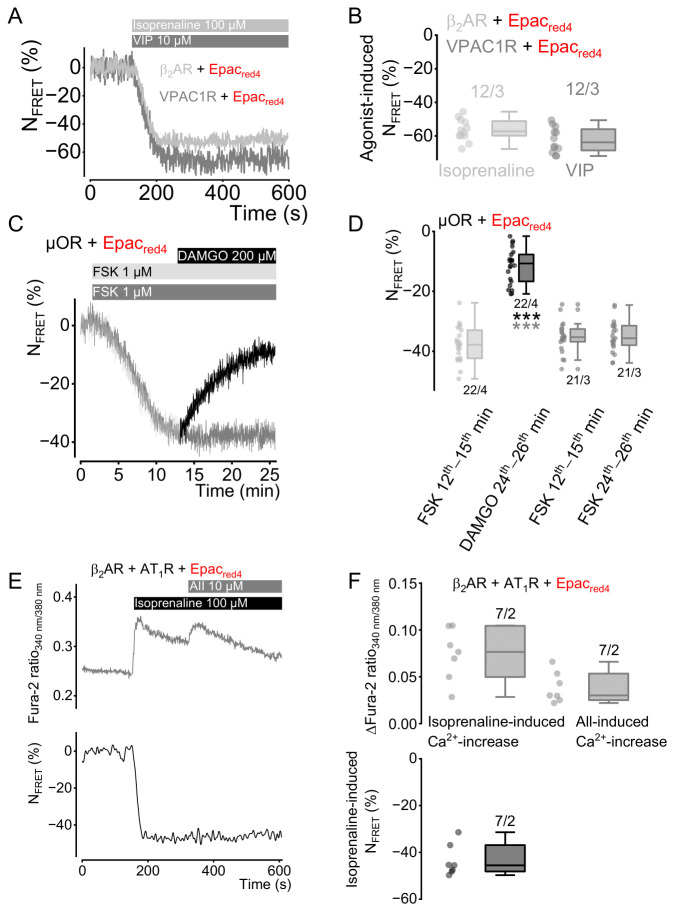
Validation of Epac_red4_ for monitoring G_s_ and G_i/o_ protein-coupled receptor signaling and multiplexing with Ca^2+^. (**A**) Representative N_FRET_ traces of HEK293T cells co-expressing Epac_red4_ and either the G_s_ protein-coupled β_2_-adrenergic receptor (β_2_AR, light gray) or the vasoactive intestinal peptide receptor 1 (VPAC1R, dark grey). Maximal stimulation was induced by bath application of isoprenaline (100 µM) or VIP (10 µM), respectively. (**B**) Summary of the maximal agonist-induced N_FRET_ decreases. Data are shown as box-and-whisker plots (median, interquartile range, and min/max whiskers) with individual data points. The numbers above each box represent the total number of measured cells and the number of independent transfections. (**C**) Representative N_FRET_ traces showing G_i/o_ protein-mediated signaling. Intracellular cAMP was first elevated by the application of 1 µM forskolin (FSK). The light grey segment of the trace represents the FSK-induced N_FRET_ decrease to the stable plateau at approximately −40%. The subsequent activation of the µ-opioid receptor (µOR) with 200 µM DAMGO (indicated by the transition to the black segment of this trace) resulted in a robust recovery of the FRET signal toward the baseline, while the application of FSK alone (grey trace) resulted in stable N_FRET_ values. (**D**) Summary of the N_FRET_ signals in response to G_i/o_ protein-coupled receptor activation selected during the forskolin-induced plateau (light grey box, 12–15 min), the subsequent DAMGO-induced recovery phase (dark grey box, 24–26 min) and during the forskolin-induced plateau without receptor stimulation (grey boxes, 12–15 min and 24–26 min). Data are presented as box-and-whisker plots (median, interquartile range, and min/max whiskers) with individual data points plotted to the left of each box. Black asterisks indicate a significant N_FRET_ increase within the same cells compared to the FSK plateau (paired Wilcoxon signed-rank test, *** *p* < 0.001). Grey asterisks indicate a significant difference between the DAMGO-stimulated group and the time-matched control group of cells stimulated with FSK alone (Mann–Whitney U-test, *** *p* < 0.001). The numbers below each box represent the total number of measured cells and number of independent transfections. (**E**) Representative traces of simultaneous cAMP and Ca^2+^ measurements in HEK293T cells co-expressing the β_2_-adrenoceptor (β_2_AR), the angiotensin II type 1 receptor (AT_1_R), and Epac_red4_. Intracellular Ca^2+^ dynamics were monitored using the ratiometric indicator Fura-2 (upper panel), while cAMP levels were recorded using Epac_red4_ (lower panel). Sequential stimulation with 100 µM isoprenaline and 10 µM angiotensin II (AII) highlights the dual G_s_/G_q_ protein signaling signature of the β_2_AR and the G_q_ protein-specific response of the AT_1_R without spectral interference. (**F**) Summary of the Ca^2+^ and cAMP responses. The upper panel shows the maximal increase in the Fura-2 ratio induced by isoprenaline and after subsequent AII application (“All-induced”). The increase was calculated as Δ (peak minus the pre-peak baseline of the Fura-2 ratio). The lower panel shows the maximal isoprenaline-induced N_FRET_ decrease. Data are presented as box-and-whisker plots (median, interquartile range, and min/max whiskers) with individual data points plotted to the left of each box. The numbers above each box represent the total number of measured cells and number of independent transfections.

To evaluate the versatility and sensitivity of the lead red-shifted cAMP sensor, we performed live-cell imaging in HEK293T cells expressing various G protein-coupled receptors (GPCRs). First, we tested the sensor’s ability to detect G_s_ protein-mediated cAMP increases upon maximal stimulation of different receptor classes. Cells expressing either the Class A β_2_-adrenergic receptor (β_2_AR) or the Class B vasoactive intestinal peptide receptor 1 (VPAC1R) were stimulated with their respective agonists. As shown in the representative traces (Figure 5A), application of 100 µM isoprenaline (for β_2_AR) or 10 µM vasoactive intestinal peptide (VIP for VPAC1R) induced a rapid and robust decrease in N_FRET_, reflecting a pronounced rise in intracellular cAMP levels. The statistical analysis (Figure 5B) confirmed consistent maximal responses achieved with both receptors, with an agonist-induced N_FRET_ change of approximately −46% to −72%. These data demonstrate that Epac_red4_ effectively monitors G_s_ protein activation across different GPCR families with a high dynamic range.

Next, we investigated whether the sensor could reliably detect G_i/o_ protein-coupled receptor-mediated decreases in cAMP levels—a significantly more challenging task than monitoring G_s_ protein activation. This difficulty arises from the stringent signal-to-noise requirements necessary to resolve inhibitory responses against a pre-established cAMP plateau. To evaluate if our lead red-shifted sensor matches the performance of the established blue-light-shifted benchmark Epac_H187_ in this demanding context [10], we performed live-cell imaging in HEK293T cells co-expressing the μ-opioid receptor (μOR) and Epac_red4_ sensor.

To establish the necessary high cAMP baseline, cells were first stimulated with 1 µM forskolin in the absence of PDE inhibitors [10,31]. As shown in the representative traces (Figure 5C), forskolin application induced a robust decrease in N_FRET_, reaching a stable plateau at approximately −40% suggesting steady-state conditions. Once this plateau was established (Figure 5C, represented by the light pink segment of the trace), application of the selective μOR agonist DAMGO (200 µM) after 12 min triggered a rapid and significant increase in N_FRET_, effectively reversing the forskolin-induced signal change toward the initial pre-stimulus baseline (Figure 5C, indicated by the transition to the dark pink segment). This upward shift reflects the G_i/o_ protein-mediated inhibition of adenylyl cyclase and the subsequent decline in intracellular cAMP levels. In contrast, control cells treated with forskolin alone (Figure 5C, grey trace) remained at the sustained FRET plateau throughout the recording, confirming that the observed signal reversal was specifically driven by μOR activation. Statistical quantification (Figure 5D) further underlines the sensor’s capability to resolve these inhibitory nuances. Following the forskolin-induced plateau (median ± SD: −38 ± 6%), the subsequent application of DAMGO triggered a significant N_FRET_ increase of +29 ± 7% (median ± SD), effectively shifting the signal to a final state of −11 ± 6% (median ± SD). This G_i/o_ protein-mediated recovery was highly significant compared to both the preceding forskolin plateau (Wilcoxon signed-rank test, *p* < 0.001; black asterisks) and the time-matched forskolin control group at 24–26 min (Mann–Whitney U test, *p* < 0.001; grey asterisks). These results demonstrate that Epac_red4_ possesses the high sensitivity and baseline stability required to monitor even subtle G_i/o_ protein-mediated inhibitory signals, successfully overcoming the inherent technical challenges of inhibitory cAMP imaging against a high-stimulus background. Altogether, these findings elevate Epac_red4_ beyond a simple detection tool, establishing it as a high-precision red-shifted benchmark capable of resolving the full complexity of bidirectional cAMP signaling from potent G_s_ protein-driven stimulation to the nuanced and precise detection of G_i/o_ protein-mediated inhibition in real-time.

### 3.7. Simultaneous Multiplexed Imaging of cAMP and Ca^2+^ Dynamics

To further demonstrate the versatility of Epac_red4_ for multi-parameter imaging, we performed simultaneous recording of intracellular cAMP and Ca^2+^ dynamics. HEK293T cells were co-expressing the β_2_-adrenoceptor (β_2_AR), the angiotensin II type 1 receptor (AT_1_R), and the Epac_red4_ sensor. By utilizing the ratiometric Ca^2+^ indicator Fura-2, we could monitor both second messengers in real-time with minimal spectral overlap (Figure 5E).

Upon stimulation with 100 µM isoprenaline, we observed an immediate increase in the Fura-2 ratio alongside the expected decrease in N_FRET_, indicating a rapid increase in intracellular Ca^2+^ concentration. While the β_2_AR primarily couples to the G_s_ protein, the observed Ca^2+^ mobilization eventually results from agonist-dependent G_q_ protein coupling of the overexpressed receptor, a mechanism recently highlighted by De Pascali et al. (2024) [31]. Subsequent application of 10 µM angiotensin II (AII) triggered a second Ca^2+^ transient via the G_q/11_ protein-coupled AT_1_R without further altering the established cAMP plateau (Figure 5E). Statistical analysis (Figure 5F) shows that using Epac_red4_ a median isoprenaline-induced N_FRET_ decrease of approximately 45% was achieved. Furthermore, sequential stimulation with isoprenaline and AII gave rise to two transient calcium increases that were not significantly different. These findings highlight that Epac_red4_ is an ideal tool for multiplexed signaling assays, allowing for the precise, real-time dissection of the interplay of cAMP and Ca^2+^ and the identification of complex GPCR signaling signatures, such as the dual G_s_/G_q_ protein coupling of the β_2_AR.

### 3.8. Characterization of PDE-Mediated cAMP Degradation Kinetics and Homeostatic Robustness

**Figure 6:**
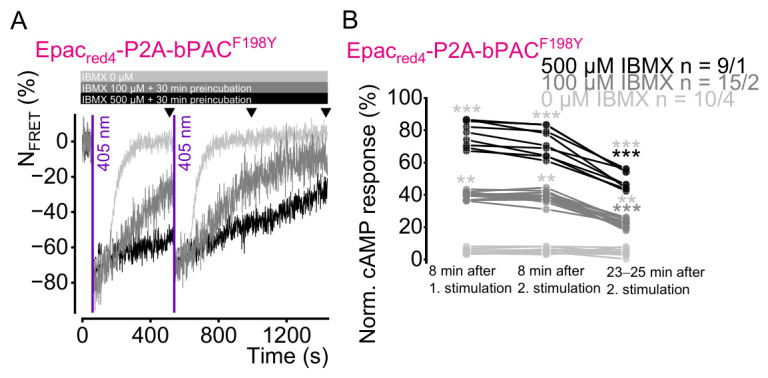
Impact of PDE inhibition on optogenetically triggered cAMP dynamics. (**A**) Representative N_FRET_ traces showing repetitive cAMP induction in HEK293T cells expressing Epac_red4_-P2A-bPAC^F198Y^. Two 405 nm laser pulses with 50% light intensity (vertical purple lines) were applied to demonstrate repeatability of the response and cumulative signaling. Cells were either untreated (0 µM IBMX, gray) or preincubated with 100 µM (gray) or 500 µM IBMX (black). (**B**) Summary of normalized cAMP responses subsequent to laser light-induced cAMP production (100%). Individual data points with color-coded lines represent normalized cAMP responses of individual cells at the indicated time points. Sample sizes, such as the total number of analyzed cells and number of independent transfections, are indicated in the panel. Light gray asterisks indicate significant differences in the IBMX-treated groups compared to the time-matched control group in the absence of IBMX (** *p* < 0.01; *** *p* < 0.001; Kruskal–Wallis test followed by post hoc Dunn’s test). Gray and black asterisks indicate significant differences within the respective treatment group between the final time point (23–25 min) and the initial stimulation (8 min after 1st stimulation) (** *p* < 0.01; *** *p* < 0.001; Friedman test followed by post hoc Dunn’s test).

To investigate the role of endogenous PDEs and the stability of cAMP homeostasis, we utilized a construct expressing the Epac_red4_ sensor and the blue-light-activated adenylyl cyclase bPAC^F198Y^ linked by a P2A self-cleaving peptide (Figure 6). This approach allows for precise, repeated, non-invasive elevations of cAMP via 405 nm laser light pulses while simultaneously monitoring the decay kinetics driven by PDE activity to probe the ability of the cell to restore cAMP equilibrium.

In control cells in the absence of the broad-spectrum PDE inhibitor 3-isobutyl-1-methylxanthin (IBMX) (0 µM IBMX, gray trace), a brief 405 nm light pulse induced a rapid decrease in N_FRET_, followed by a rapid recovery to the pre-stimulus baseline within approximately 400 s (Figure 6A). This recovery phase reflects the kinetics of high basal PDE activity, which serves as the primary engine of the cellular homeostatic machinery. Crucially, this restoration of cAMP homeostasis was highly repeatable. The second light stimulus exhibited nearly identical N_FRET_ amplitudes and kinetics with a full recovery to the pre-stimulus baseline. This demonstrates that under physiological conditions, the cell possesses a robust capacity to “reset” its cAMP levels, ensuring that subsequent signaling events are not masked or distorted by previous activity. To quantify how impaired PDE activity affects these dynamics, cells were preincubated for 30 min with either 100 µM or 500 µM IBMX. As shown in Figure 6A, while the initial activation kinetics (the rapid FRET drop) remained intact upon each light pulse, the recovery N_FRET_ kinetics were significantly slowed or abolished. 100 µM IBMX significantly slowed the cAMP degradation kinetics, leading to a delayed return to baseline (Figure 6A, cyan trace). A concentration of 500 µM almost entirely abolished the restoration of the cAMP homeostasis (Figure 6A, teal trace). This illustrates that while the sensor remains fully responsive to repeated stimuli, the loss of homeostatic control transforms transient, pulsatile signals into a sustained and cumulative cAMP elevation.

Statistical quantification of the normalized intracellular cAMP levels (Figure 6B) confirms these observations. While control cells effectively restored the homeostatic setpoint after each stimulation, maintaining the capacity for repeatable kinetic cycles, cells treated with 500 µM IBMX exhibited a complete failure to reset. In these cells, cAMP levels remained in a state of chronic elevation after 8 min of the second stimulation (median ± SD: 71 ± 9%) and even 25 min after the final stimulation with 47 ± 6% (median ± SD). Ultimately, these findings are pointing to the view that the Epac_red4_ sensor, in combination with the P2A-linked bPAC, can serve as a sensitive cAMP detection platform and provides insights into the cellular cAMP homeostasis. Furthermore, precise dissection of the fragile kinetic equilibrium between second messenger synthesis and phosphodiesterase-mediated degradation can be monitored in real-time.

## 4. Discussion

Cellular signaling relies on the intricate interplay between second messengers, with Ca^2+^ and cAMP acting as ubiquitous and central regulators [32], embedded within a broader network of key mediators including IP_3_, DAG, and cGMP that collectively orchestrate cellular viability. Rather than acting in isolation, these messengers form a tightly coupled, interdependent signaling network. As is well established, Ca^2+^ levels shape cAMP dynamics via Ca^2+^-regulated adenylyl cyclases and phosphodiesterases, while cAMP-dependent protein kinase A reciprocally modulates Ca^2+^ influx and release. This bidirectional cross-talk is essential for generating complex signaling regimes such as the synchronized oscillations in pancreatic β-cells [33,34] or synaptic plasticity in neurons [35]. Measuring only one parameter provides a fragmentary view that can lead to a fundamental misinterpretation of the cellular state. The ability to monitor Ca^2+^ and cAMP simultaneously in the same cell is therefore essential for uncovering the causal relationships and phase shifts that define physiological homeostasis. For example, in pancreatic β-cells, the precise timing between Ca^2+^ oscillations and cAMP transients determines insulin secretion [36], while in neurons, coordinated Ca^2+^/cAMP dynamics underlie the induction of long-term potentiation and synaptic plasticity [37,38].

Despite the scientific demand, multiparametric imaging of cAMP alongside Ca^2+^ has been hindered by significant physical and optical constraints. Traditional CFP/YFP-based FRET sensors occupy the most vital part of the visible spectrum and create massive spectral overlap with established green Ca^2+^ indicators like the GCaMP family, making simultaneous imaging challenging. Likewise, the combination of CFP/YFP-based FRET sensors with the synthetic dye Fura-2 is problematic due to overlapping fluorescence emissions [39]. While the mOrange2-mCherry FRET pair has been successfully implemented to enable dual-FRET imaging in complex live-cell contexts, for example, concurrent Src and MT1-MMP activity mapping in single cells [40], significant spectral crosstalk between mOrange2 and mCherry remains, often narrowing the effective dynamic range of the sensors [41]. Red-shifted fluorescent proteins have historically lagged behind their green counterparts in quantum yield and brightness, which can limit sensor sensitivity and necessitate higher excitation intensities that increase phototoxicity and bleaching [42]. Engineering strategies aimed at enhancing dynamic range and photostability of red FRET pairs, including self-associating domain designs, illustrate one solution to these limitations but often still fall short of the performance needed for robust multiparametric imaging [43]. Although extending the sensing range further into the near-infrared would mitigate spectral overlap with visible Ca^2+^ indicators [44], this typically entails substantial technical effort to adapt excitation sources and detection hardware, which is not commonly implemented in standard imaging setups.

Our findings suggest that Epac_red4_ showed the most robust performance among the red-shifted variants. Notably, the utilization of mOrange2 as a FRET donor in Epac_red2_ and Epac_red3_ proved less effective than the use of tdLanYFP in Epac_red4_. One reason for this might be its high pKa of 6.5 compared to the significantly more acid-stable 3.9 of tdLanYFP. This pH sensitivity is particularly pronounced in the tandem variant Epac_red3_, which exhibited twice the variance in N_FRET_ decreases under both maximally endogenous and light-induced bPAC activation. This might reflect a compounded pH dependency inherent to the doubled pKa in the tandem construct. Beyond its suboptimal acid stability, mOrange2 is further constrained by a lower quantum yield of 0.6 and a brightness of 34.8. These values fail to match the superior performance of tdLanYFP, which offers a quantum yield of 0.92 and a brightness of 122.4. While the mOrange2-mCherry pair was successfully implemented in complex imaging scenarios, significant crosstalk remains a major drawback that narrows the dynamic range of the sensor.

In contrast, tdScarlet3 represents a substantial technological advancement with an exceptional quantum yield of 0.70 and a brightness nearly three times that of mCherry2. This significantly enhances the signal-to-noise ratio in the FRET channel. The excitation maximum of mScarlet3 at 569 nm provides a superior spectral overlap with the mOrange2 emission compared to mCherry2. This results in a larger Förster radius and increased energy transfer efficiency. Coupled with its superior acid stability, tdScarlet3 is far superior as an acceptor for robust biosensor design. Provided that the quantum yield and pKa of mOrange2 could be further optimized in a next-generation “mOrange3”, a sophisticated dual-FRET architecture would become possible. Pairing an optimized mOrange3 with tdScarlet3 would establish a system that exhibits minimal spectral excitation overlap with established mTq2-tdVenus FRET pairs.

This streamlined approach facilitates the simultaneous recording of cAMP dynamics in distinct subcellular nanodomains via targeted Epac sensors. Furthermore, this strategy allows for the integration of modern mTurquoise2-cpmVenus Cameleon sensors for ratiometric calcium imaging alongside mOrange3-based cAMP sensors, which offers a powerful and purely genetically encoded alternative to traditional Fura-2-based multiplexing. The addition of the blue-light-activated adenylyl cyclase bPAC^F198Y^ transforms this observational setup into a causal platform. Beyond demonstrating spectral compatibility, this all-optical configuration enables temporally precise perturbation of cAMP levels. Furthermore, we could show that Epac_red4_ can be particularly useful for analyzing recovery kinetics and phosphodiesterase-dependent signal shaping (Figure 6). However, future studies with targeted bPAC pulses combined with subtype-specific PDE inhibitors might enable a deeper understanding of how specific enzyme isoforms guard individual nanodomains.

The practical utility of Epac_red4_ is most notably demonstrated by its seamless integration into multiplexed imaging protocols. While traditional blue-shifted sensors often restrict the available spectral space, the red-shifted profile of Epac_red4_ enables the use of the ratiometric Ca^2+^ indicator Fura-2 with minimal spectral interference. This is a critical advantage, as Fura-2 remains a gold standard for quantitative Ca^2+^ imaging due to its ratiometric nature, yet it was previously difficult to combine with CFP/YFP-based cAMP sensors. Our simultaneous recordings of cAMP and Ca^2+^ (Figure 5E,F) reveal the power of this combination in dissecting complex GPCR signaling signatures. The observation of a rapid Ca^2+^ transient following β_2_AR activation—typically regarded as a G_s_ protein-coupled event—highlights the ability of this GPCR to capture nuanced signaling crosstalk. As recently elucidated by De Pascali et al. (2024) [31], the β_2_AR can promote agonist-dependent G_q_ protein coupling, particularly in overexpression systems. By resolving both the G_s_ protein-driven cAMP rise and the G_q_ protein-mediated Ca^2+^ mobilization in the same cell, Epac_red4_ provides a comprehensive readout of receptor pleiotropy. The fact that subsequent AT_1_R activation triggered a further Ca^2+^ peak without perturbing the established cAMP plateau further underlines the independence and robustness of our dual-parameter setup. This capability is essential for studying “signal-tuning”, where multiple G protein pathways converge to shape the final cellular response. Future experiments investigating endogenously expressed receptors in native cells using multiplexing approaches might help unravel the complex interplay between cAMP and Ca^2+^ signaling.

Besides spectral properties and dynamic range, the cAMP affinity is an important criterion when selecting a genetically encoded cAMP biosensor. Epac_red4,_ which is based on Epac_H187,_ retains the Epac sensing domain, including the Q270E mutation, previously reported to decrease the K_d_ from approximately 9.5 µM to 4.0 µM, thereby increasing cAMP affinity by about 2.5-fold [11]. Since the cAMP-binding domain of Epac_red4_ was not modified, its cAMP-binding affinity is expected to remain comparable to that of Epac_H187_, with an apparent K_d_ in the single-digit micromolar range. However, because the fluorescent protein pair was redesigned, the exact K_d_ of Epac_red4_ cannot be inferred directly and must be determined experimentally. Indeed, changes in the fluorescent proteins may alter the conformational coupling within the biosensor and thereby affect its apparent affinity and overall sensor properties.

Recently developed genetically encoded cAMP biosensors exhibit a broad range of apparent cAMP affinities and employ different optical readout principles. Among currently available sensors, apparent K_d_ values range from the hundreds of nanomolar range to the single-digit micromolar range. cAMPinG1 [17] and R-FlincA [16] exhibit apparent K_d_ values in the hundreds of nanomolar range, whereas G-Flamp1 [14], Flamindo2 [13], Pink Flamindo [15], gCarvi [18], and Epac-based FRET sensors [11] including cAMPFIRE [19] operate in the single-digit micromolar range. Sensors in the hundreds of nanomolar range are particularly well suited for detecting small or basal changes in cAMP but may approach saturation during strong receptor stimulation or pharmacological elevation of cAMP. In contrast, sensors operating in the single-digit micromolar range provide a broader working range for monitoring large cAMP elevations and their subsequent recovery. Thus, the optimal biosensor depends on the expected intracellular cAMP concentrations and the biological question being addressed.

In addition to affinity, the optical design of a biosensor is an important consideration. Single-fluorescent-protein sensors, including cAMPinG1 [17], G-Flamp1 [14], Flamindo2 [13], Pink Flamindo [15], and R-FlincA [16], require relatively simple imaging setups, whereas Epac_H187_, cAMPFIRE, and Epac_red4_ are FRET-based biosensors that provide internally normalized ratiometric readouts [11,19]. gCarvi represents an alternative ratiometric design based on the bacterial cAMP receptor protein [18]. However, currently optimized Epac-derived FRET sensors largely rely on cyan/yellow fluorescent protein pairs, limiting their compatibility with blue-light optogenetic actuators such as bPAC and UV-excited indicators such as Fura-2. Epac_red4_ addresses this limitation by replacing the conventional cyan/yellow FRET pair with a yellow/red FRET pair while preserving ratiometric detection, thereby enabling quantitative multiparameter imaging with substantially reduced spectral overlap.

To summarize, our findings solidify Epac_red4_ as a premier high-fidelity benchmark that transcends simple detection and provides a high-resolution window into the fundamental robustness of cellular homeostasis. Furthermore, our findings identify Epac_red4_ as a robust red-shifted cAMP sensor that combines quantitative FRET readout with spectral compatibility for optogenetic stimulation and simultaneous multiparameter imaging. Future studies should determine how well Epac_red4_ performs in more physiologically relevant systems and whether it can be adapted to resolve highly localized cAMP nanodomains in subcellular compartments.

## Figures and Tables

**Table 1:** PCR primers utilized for site-directed mutagenesis and molecular cloning. The table summarizes the primers employed for fluorophore extractions, deletions, and the introduction of specific point mutations (e.g., bPAC^F198Y^). For each modification, the primer designation, nucleotide sequence in 5′ to 3′ orientation, and the optimized annealing temperature (T_a_) are provided. Lowercase letters within the sequences indicate introduced modifications, such as restriction sites or linker sequences.

Modification/Target	Primer Designation	Sequence (5′–3′)	T_a_ (°C)
H187 with deletion of mTq2	H187_Del mTq2_f	agcccgtgcagctgcccggcGATATCAGCCCGTGGGAACTCATG	70.5
	H187_Del mTq2_r	GGTGGCGGCAAGCTTGGG	
Extraction of cpmCherry2	ExcpmCh2_f	gacccaagcttgccgccaccATGGCCTACAACGTCGACATCAAGTTGGACATC	72
	ExcpmCh2_r	GCCGGGCAGCTGCACGGG	
tdV-Epac-cpmCh2 with deletion of tdV	Del tdV_f	tggacgagctgtacaagtaaAATTCCCTCGAGGTTAACGC	65.7
	Del tdV_r	GCTAGCTGGCTCCAGCTC	
Extraction of mOrange2	ExmOr2_f	gagagctggagccagctagcATGGTGAGCAAGGGCGAG	65.1
	ExmOr2_r	TTACTTGTACAGCTCGTCCATG	
Deletion of cpmCherry2	cpmCherry2Del_f	CGATATCAGCCCGTGGGAAC	69
	cpmCherry2Del_r	GGTGGCGGCAAGCTTTCC	
Deletion of tdVenus	tdVenDel_f	TCTAGAGGGCCCTATTCTATAGTG	67
	tdVenDel_r	GCTAGCTGGCTCCAGCTC	
Insertion of EcoRI in Epac	EPAC-insEcoRI_f	gaattcGCCCTATTCTATAGTGTCACC	63
	EPAC-insEcoRI_r	CCTCTAGAGCTAGCTGGC	
Deletion of LiefAct tag and Insertion of NheI in mScarlet3	mSc3_insNheI_f	agcGCCACCATGGATAGCACC	64
	mSc3_insNheI_r	agcGGTGGAATTCGAAGCTTGAG	
Insertion of linker in Nhe1-mScarlet3	mSc3_inlinker_f	gggctcctcaggagaggaggataacTAACTGTACAAGTAAAGCGGCCGCGAC	73
	mSc3_inslinker_r	gagcccgtagaaccagtgccagtcgaGGAGCCACCGGAGCCG	
Opening of Nhe1-mScarlet3-linker	mSc3-linker_Split_f	TAACTGTACAAGTAAAGCGGCCG	68
	mSc3-linker_Split_r	gttatcctcctctcctgaggagc	
Extraction of mScarlet3-EcoRI	mSc3-EcoRI_f	gatagcaccgaggcagtgatcaa	70
	mSc3-EcoRI_r	taaggagccaccggagcc	
Insertion of Codon optimized ends in mOrange2	mOr2codop_f	ATGGTTTCAAAGggcgaggagaataacatggc	67
	mOr2codop_r	TTTATAGAGTTCATCcatgccgccggtgga	
Insertion of linker in mOrange2	mOr2inslinker_f	gggctcctcaggagaggaggataacTAAGCGGCCGCGACTCTA	67
	mOr2inslinker_r	gagcccgtagaaccagtgccagtcgaCTTGTACAGCTCGTCCATGC	
Opening of mOrange2-linker	mOr2 linker Split_f	GCGGCCGCGACTCTAgat	69
	mOr2 linker Split_r	gagcccgtagaaccagtgccagtcgaGGAGCCACCGGAGCCG	
Extraction of mOrange2 (cod opt ends)	Ex mOr2codoptends_f	ATGGTTTCAAAGggcgaggagaataacatggc	67
	Ex mOr2codoptends_r	TTTATAGAGTTCATCcatgccgccggtgga	
Extraction tdOrange2	Ex tdOr2_f	tcgccaccatggtgagcaa	69
	Ex tdOr2_r	TTTTATAGAGTTCATCcatgccgccg	
Extraction tdLanYFP	Ex tdLanYFP_f	tagccaccATGGTCTCCAAAGGAGAGG	64
	Ex tdLanYFP_r	tCTTGTACAGCTCGTCCATG	
Exchange of F198Y in bPAC	bPAC F198Y_f	AGTGACCAAGtatATCGGCGACTGCG	70
	bPAC F198Y_r	TCGCCGCCGTAGGCG	
Deletion of IRES	IRES del_f	Atgatgaagcggctggtgtac	60
	IRES del_r	cgtGAATTCggcggagccaccg	

**Table 2:** Molecular architecture and photophysical properties of the Epac-based cAMP FRET sensors used in this study. The table summarizes the molecular composition of the established blue-shifted reference sensor (Epac_H187_) and the four newly developed red-shifted variants (Epac_red1_–Epac_red4_). Excitation wavelengths (nm) indicate the specific laser lines utilized for FRET donor and FRET acceptor excitation, respectively. Theoretical FRET efficiencies were calculated as the product of the Förster radius (R_0_) and the quantum yield of the acceptor (QY_A_) using the FPbase FRET calculator (https://www.fpbase.org/fret/ (accessed on 15 January 2026)). Epac: Exchange protein directly activated by cAMP, mTq2: monomeric Turquoise2, tdV: tandem dimer of circularly permuted monomeric Venus, cpmCh2: circularly permuted monomeric Cherry2, mOr2: monomeric Orange2, tdOr2: tandem dimer Orange2, tdSc3: tandem dimer Scarlet3, tdLanYFP: tandem dimer LanYFP. The colors denote the individual cAMP sensor constructs and are used as a sensor-specific color code in Figure 1 and Figure 2.

	Construct	Designation	Excitation (nm)	FRET Efficiency (R_0_ × QY_A_)
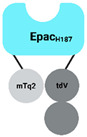	**mTq2-** **Epac-** **tdV**	**Epac_H187_**	**405** **/** **515**	**37**
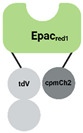	**tdV-** **Epac-cpmCh2**	**Epac_red1_**	**515** **/** **594**	**13**
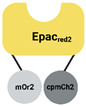	**mOr2-** **Epac-cpmCh2**	**Epac_red2_**	**515** **/** **594**	**14**
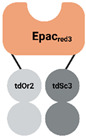	**tdOr2-** **Epac-** **tdSc3**	**Epac_red3_**	**515** **/** **594**	**47**
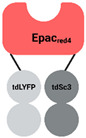	**tdLanYFP-Epac-** **tdSc3**	**Epac_red4_**	**515** **/** **561**	**50**

## Data Availability

All data reported in the paper will be shared by the corresponding authors upon request.

## Supplementary Materials

The following supporting information can be downloaded at: https://www.mdpi.com/article/10.3390/cells15131223/s1, Material and Methods; Figure S1: Spectral characterization of the fluorescent proteins used in this study; Figure S2: Spectral analysis and functional characterization of the cAMP FRET sensors. Table S1: Spectral acquisition settings for live-cell fluorescence measurements; Table S2: Spectral characteristics of the fluorescent proteins utilized in this study.

## Abbreviations

The following abbreviations are used in this manuscript:

Epac	Exchange proteins directly activated by cAMP
FRET	Förster resonance energy transfer
bPAC	bacterial photoactivated adenylyl cyclase
cAMP	Cyclic adenosine monophosphate
