# Supplementary material for: Red-Shifted Epac-Based FRET cAMP Sensors for All-Optical cAMP Control and Multiparameter Imaging
Source: Cells. 2026 Jul 6;15(13):1223. doi: 10.3390/cells15131223 (PMC13360413; doi:10.3390/cells15131223)
Supplement: Supplementary file 1 [file cells-15-01223-s001.zip › cells-4305631-supplementary.pdf]

Supplementary Information

Material and Methods

Live-cell fluorescence excitation and emission spectroscopy

HEK293T cells were transiently transfected with 1-2 µg of the respective fluorescent protein or FRET sensor using the non-lipid-based transfection reagent GeneJuice® (Merck Millipore, Schwalbach, Germany) according to the manufacturer's instructions. Spectroscopic measurements were performed 48–72 h after transfection. Approximately 16–30 h before measurements, transfected cells (45,000 cells or 35,000 cells per well) were detached and reseeded into poly-L-lysine-coated glass-bottom 96-well plates (0.1 mg/mL; Cat. No. P-1524, Sigma-Aldrich; Corning Costar 3610; growth area 32 mm^2, well diameter 6.35 mm). Cell seeding density was adjusted according to the expected incubation period, resulting in approximately 50,000 cells per well at the time of measurement while avoiding overconfluence during the experimental period. Cells were maintained in phenol-red-free, HEPES-buffered DMEM (Cat. No. 11594416, Gibco, Life Technologies, Carlsbad, CA, USA). Excitation and emission spectra were recorded in bottom-read mode using a CLARIOstar multimode plate reader (BMG LABTECH). Spectral data were analyzed using MARS software (BMG LABTECH, Ortenberg, Germany) and OriginPro 2026 (OriginLab). Background fluorescence was determined from untransfected HEK293T cells measured under identical conditions and subtracted from all recordings. Thus, all spectra presented in the figures represent background-corrected difference spectra. All spectra were acquired with a wavelength increment of 1 nm and an optical bandwidth of 8 nm, with excitation and emission spectra recorded across the specified spectral ranges. For FRET sensor measurements, spectra were recorded under basal conditions and after stimulation with 1 mM forskolin and 500 µM IBMX. Following compound addition, cells were incubated for 5 min before acquisition of stimulated spectra. Because excitation and emission characteristics differed substantially among the investigated fluorescent proteins and FRET sensors, acquisition parameters were optimized individually for each construct. The corresponding settings are summarized in Supplementary Table S1.

Supplementary Table S1. Spectral acquisition settings for live-cell fluorescence measurements. Construct-specific excitation and emission scan ranges, detection wavelengths, optical bandwidths, and detector gain settings used for fluorescence spectroscopy in living HEK293T cells. Spectral scans were performed using a CLARIOstar multimode plate reader with an optical bandwidth of 8 nm and a wavelength increment of 1 nm. Optical bandwidths are reported as half-width values (± nm) around the respective center wavelength. All spectra shown in the figures represent background-corrected difference spectra obtained by subtracting signals from untransfected HEK293T cells measured under identical conditions.

Construct	Excitation scan (nm)	Detection window (nm)	Emission scan (nm)	Excitation window (nm)	Gain Ex	Gain Em
mTurquoise2	350–451	520 ± 50	457–670	428 ± 10	2000	2300
tdVenus/tdLanYFP	380–601	670 ± 50	520–670	492 ± 10	2200	1200
mOrange2/tdOrange2/tdScarlet3/cpmCherry2	380–611	680 ± 50	520–670	492 ± 10	1600	1600
$Epac_{H187}$	380–601	670 ± 50	460–670	435 ± 6	2400	1600
$Epac_{red1-4}$	380–601	670 ± 50	520–670	492 ± 10	2000	2000

Supplementary Table and Figures

Supplementary Table S2. Spectral characteristics of the fluorescent proteins utilized in this study. The table provides the experimental peak maxima of the indicated fluorescent proteins. Mean values are derived from the number of measurements (n) indicated. Standard deviation (SD) reflects the variability of peak detection within our setup. For comparison, reference values were obtained from the FPbase database (https://www.fpbase.org/). Abbreviations: Ex, excitation; Em, emission. All units are given in nanometers (nm).

Fluorescent protein variant and Mode	n	Mean Peak (nm)	± SD (nm)	FPbase reference maximum (nm)
mTq2 Ex	24	432.8	10.2	434
mTq2 Em	24	476.5	2.1	474
tdVenus Ex	12	515.5	0.8	515
tdVenus Em	12	538.8	0.4	527
tdLanYFP Ex	9	511.7	0.9	513
tdLanYFP Em	12	530.0	0.0	531
mOr2 Ex	36	551.4	1.5	549
mOr2 Em	36	566.1	1.4	565
tdOr2 Ex	36	551.6	1.6	549
tdOr2 Em	24	566.4	1.1	565
cpmCh2 Ex	35	573.9	1.9	589
cpmCh2 Em	36	608.1	9.1	610
tdSc3 Ex	10	567.8	0.6	569
tdSc3 Em	10	594.3	1.1	592

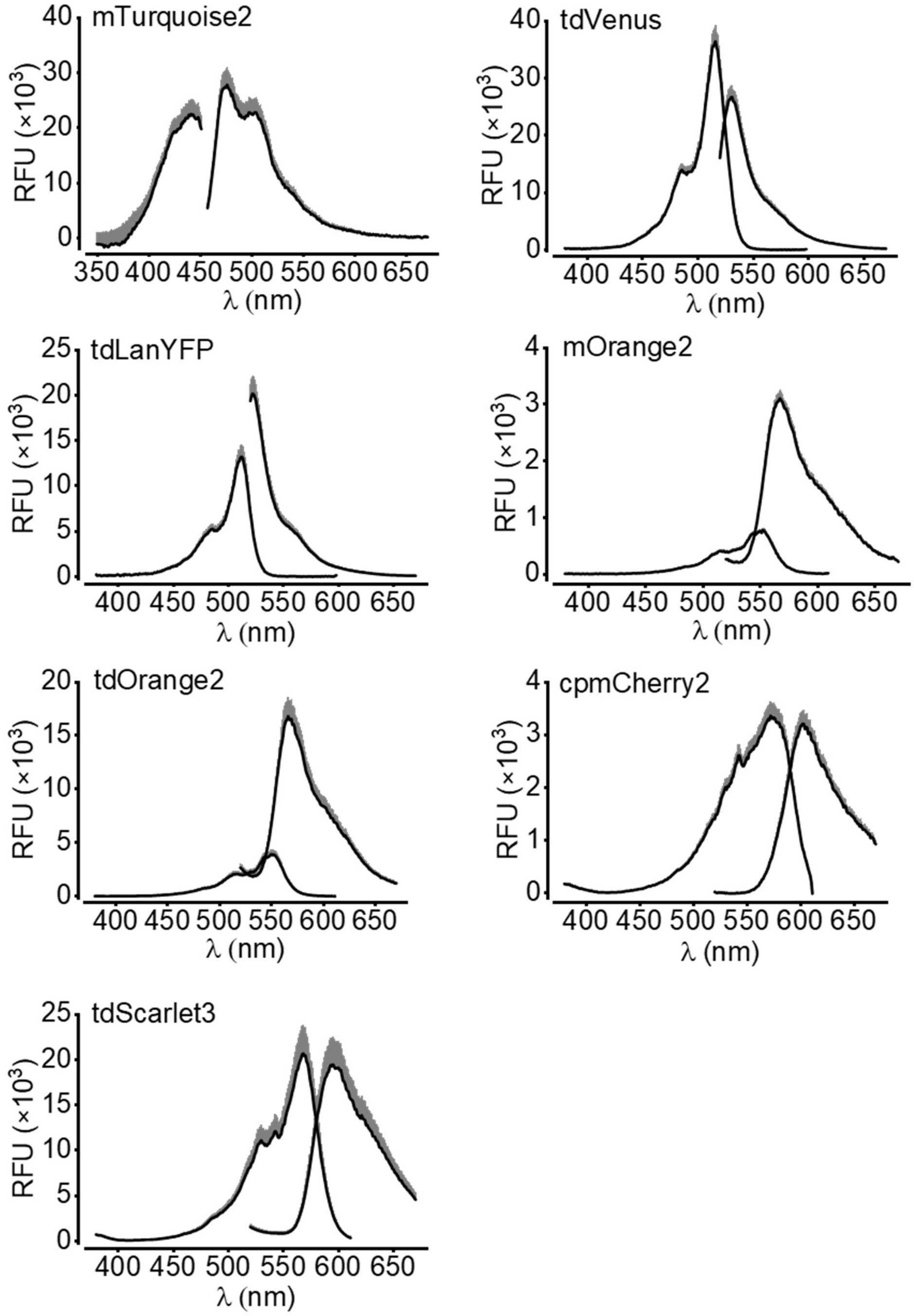

Supplementary Figure S1. Spectral characterization of the fluorescent proteins used in this study. Representative background-corrected excitation (left curves) and emission (right curves) spectra of the indicated fluorescent proteins transiently expressed in living HEK293T cells. All spectra represent background-corrected difference spectra, calculated by subtracting the autofluorescence signals of untransfected HEK293T cells measured under identical conditions. Data are presented as the mean (solid line) ± SEM (shaded area) derived from n=12 biological replicates. Detailed spectral acquisition settings are provided in Supplementary Table S1.

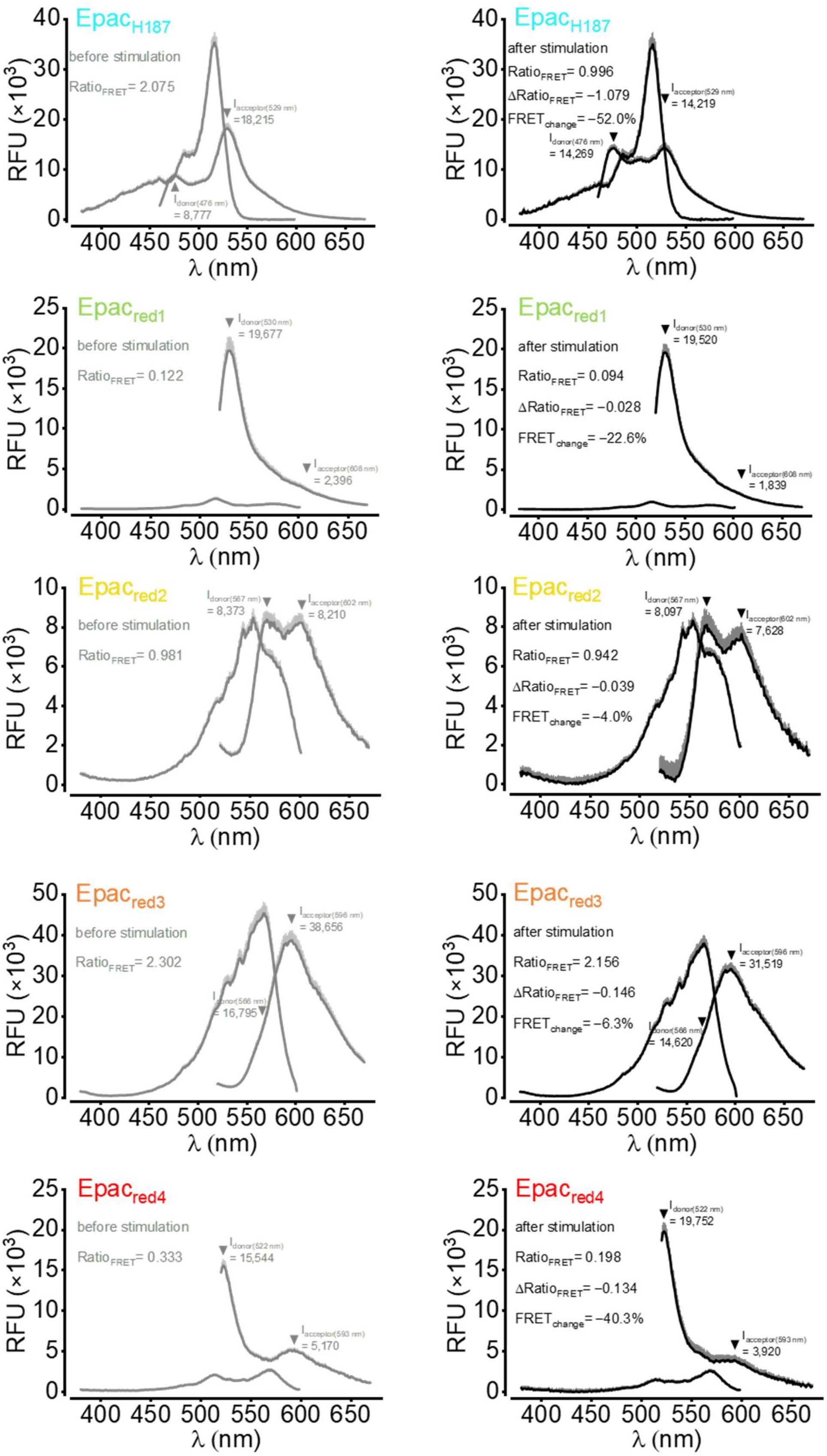

Supplementary Figure S2. Spectral analysis and functional characterization of the cAMP FRET sensors. Representative excitation (left curves) and emission (right curves) spectra of the indicated FRET sensors recorded in live HEK293T cells. Left panels: Spectra recorded under basal conditions. Right panels: Spectra recorded after maximal cAMP elevation induced by incubation with 1 mM forskolin and 500 µM IBMX. Arrows denote the peak

fluorescence intensities at the donor and acceptor emission maxima. In the case of $Epac_{red1}$ an acceptor emission maximum was not observed and thus, estimated emission maximum of cpmCherry2 was used. In case of $Epac_{red3}$ a donor emission maximum was not observed and therefore, estimated emission maximum of tdOrange2 was used. All traces represent background-corrected difference spectra, calculated by subtracting the autofluorescence signals of untransfected HEK293T cells from the raw data. Data are presented as the mean (solid lines) ± SEM (shaded areas) derived from n = 12 biological replicates. The $Ratio_{FRET}$ was calculated as the ratio of acceptor emission intensity ($I_{acceptor}$) to donor emission intensity (I_{donor}) at the respective peak wavelengths. $\Delta Ratio_{FRET}$ was calculated as $Ratio_{FRET}$ before and after stimulation. $FRET_{change}$ was calculated as $\Delta Ratio_{FRET}$ divided by $Ratio_{FRET}$ before stimulation in %. $\Delta Ratio_{FRET}$ and $FRET_{change}$ indicate the sensor response upon cAMP increase. The calculated values represent uncorrected, 'raw' FRET ratios ($Ratio_{FRET}$) derived from background-subtracted emission spectra without correction of bleed-through and cross-excitation. The calculated relative spectral FRET changes ($FRET_{change}$) reproduced the performance ranking obtained from live-cell N_{FRET} imaging, with $Epac_{red4}$ exhibiting the largest dynamic response following cAMP stimulation.
